# Ruxolitinib Cream Has Dual Efficacy on Pruritus and Inflammation in Experimental Dermatitis

**DOI:** 10.3389/fimmu.2020.620098

**Published:** 2021-02-15

**Authors:** Monika D. Scuron, Brittany L. Fay, Andrew J. Connell, Michael T. Peel, Paul A. Smith

**Affiliations:** Incyte Research Institute, Inflammation and Autoimmunity Department, Wilmington, DE, United States

**Keywords:** dermatitis, ruxolitinib, inflammation, pruritus, JAK/STAT signaling pathway

## Abstract

The goal of this study was to elucidate the anti-pruritic and anti-inflammatory efficacy of ruxolitinib cream in experimentally-induced dermatitis. Atopic dermatitis (AD), the most common chronic relapsing inflammatory skin disease, significantly impairs patients’ quality of life, with pruritus being a common complaint. The sensation of itch results from the interplay between epidermal barrier dysfunction, upregulated immune signaling and the activation of the central nervous system. The Janus kinase (JAK)-signal transducer and activator of transcription (STAT) pathway plays a central role in pro-inflammatory cytokine signaling in AD. Ruxolitinib cream is a potent and selective JAK1/2 inhibitor currently undergoing clinical evaluation in adults with mild-to-moderate AD (NCT03745638, NCT03920852 and NCT03745651). The efficacy of ruxolitinib cream was tested in murine models of acute and chronic dermatitis and was also characterized in an *ex vivo* human skin dermatitis model. Ruxolitinib cream was highly effective at ameliorating disease symptoms in multiple murine dermatitis models through downregulation of T helper (Th)2-driven inflammation, resulting in reduced skin thickening and decreased itch. Pathway analysis of mouse ear tissue and human skin explants underscored the role for ruxolitinib in ameliorating inflammation and reducing itch *via* modulation of the JAK-STAT pathway. Together, the data offer a strong rationale for the use of ruxolitinib cream as a potent therapeutic agent for the clinical management of atopic dermatitis.

## Introduction

Atopic dermatitis (AD) is the most common inflammatory skin disease, with intense pruritus being the major and most burdensome symptom (1, 2). Intractable pruritus has a significant impact on patients’ quality of life and constitutes one of the unmet medical needs (3, 4).

The Janus kinase (JAK) signal transducer and activator of transcription (STAT) pathway, a classical signal transduction pathway for numerous cytokines and growth factors, has been shown to play an important role in the dysregulation of immune responses in AD (5). The JAK family is comprised of four types of cytoplasmic tyrosine kinases: JAK1, JAK2, JAK3, and TYK2. The STAT family contains seven members: STAT1, STAT2, STAT3, STAT4, STAT5A, STAT5B, and STAT6 (6). Multiple immune mediators present within the inflamed skin utilize class I/II cytokine receptors and are dependent on the JAK-STAT pathway for signal transduction (7–10).

Eczematous lesions are characterized by T helper (Th)2 cells leading to eosinophil recruitment and immunoglobulin production *via* the secretion of distinct cytokines, including interleukins (IL) IL-4, IL-5 and IL-13 (11). Epithelial mediators, such as IL-33 and thymic stromal lymphopoietin (TSLP), also play an important role in the type 2 innate immune response. IL-33, constitutively produced by skin epithelial cells, binds to the ST2 receptor on Th2 and other innate immune cells, and utilizes JAK1/2 kinase activity for downstream signal transduction (12, 13). In AD patients, IL-33 overexpression in the epidermis, infiltration of ST2-positive cells and elevated serum IL-33 levels have been reported (14, 15). Transgenic mice with constitutive epidermal-specific IL-33 expression (IL-33tg) spontaneously develop a progressive, AD-like skin inflammation and pruritus (16). Moreover, the epithelial cell-derived cytokine TSLP also promotes Th2 cytokine-expressing cells (17). TSLP, signaling through JAK, acts as a dual mediator of inflammation and pruritus (18, 19).

The knowledge of JAK-STAT pathway involvement in inflammatory skin diseases has led to the development of oral and topical JAK inhibitors (5, 9, 10, 20–22) (23, 24). Novel topical selective JAK inhibitors represent a promising option in the treatment of AD (10, 25, 26), and a topical pan-JAK inhibitor was recently approved in Japan for the treatment of atopic dermatitis (27). The focus of our study was ruxolitinib cream, a potent, selective JAK1/2 inhibitor that demonstrated significant clinical benefit in a phase 2b trial in adults with AD (NCT03011892) (28, 29) and is currently being evaluated for the treatment of mild-to-moderate AD (NCT03745638, NCT03920852 and NCT03745651).

The goal of the current study was to characterize the dual anti-inflammatory and anti-pruritic potential of ruxolitinib cream in mouse models of experimentally-induced dermal inflammation. In addition to murine models of AD, the dual efficacy of ruxolitinib cream on pruritus and inflammation was assessed *ex vivo* using human skin explants.

## Materials and Methods

### Animal Experiments

Animal studies were approved by the Institutional Animal Care and Use Committee (IACUC) and performed in Assessment and Accreditation of Laboratory Animal Care (AAALAC) accredited facilities.

Female BALB/c mice were purchased from the Jackson Laboratories (USA). IL-33 transgenic (IL-33tg) mice were produced by TransGenic Inc. (Japan). All animals were housed under specific pathogen-free conditions and reared in line with standardized methods at 22 ± 1°C on a 12‐h light/dark cycle with free access to food and water.

### Acute TSLP-Induced Dermatitis

BALB/c mice were randomized to the following groups; 1) sham injected untreated, 2) vehicle cream b.i.d., 3) 1.5% w/w ruxolitinib cream b.i.d. or 4) 0.05% w/w clobetasol cream q.d. For groups 2–4, murine TSLP (Invitrogen, USA) in sterile saline (3 µg in 20 µl) was injected intradermally into the outer pinna of the right ear on days 0, 2, 4, and 7. Topical cream (20 mg) was applied to the right ear from day 0 to 9. Ear swelling was measured with a thickness gauge (Mitutoyo, Japan) at 24, 48, and 72 h post day 7 injection. At study termination, 6 mm ear punch biopsies were collected, weighed, and fixed for histopathology. RNA isolation was performed on the remaining ear skin.

In a separate TSLP-induced dermatitis study, the spontaneous activity of vehicle and 1.5% w/w ruxolitinib b.i.d. treated mice was quantified using continuous home cage video recording (Vium, USA). The Vium platform provides continuous real-time measurement of activity.

### Chronic FITC-Induced Dermatitis

BALB/c mice were randomized to the following groups: vehicle cream b.i.d., 1.5% w/w ruxolitinib cream q.d., 1.5% w/w ruxolitinib cream b.i.d. and 0.05% w/w betamethasone cream q.d. and dosed on both ears with 20 mg cream per ear for the duration of the study.

On day 0, mice were sensitized on the shaved abdomen with 100 µl of 0.5% w/v fluorescein isothiocyanate (FITC) in acetone-dibutyl phthalate 1:1 (v:v) solution (Sigma Aldrich, USA). Sensitization was repeated on days 1 and 2. On day 7 mice were challenged with 20 µl of FITC solution on the right ear. Ear re-challenge was repeated once per week for 4 additional weeks to induce chronic skin inflammation. Ear swelling was measured at 24 and 72 h post FITC challenge. At termination, ear biopsies were weighed and RNA was isolated. Right draining (auricular) lymph nodes were excised for immunophenotyping.

### Immunophenotyping

Lymph nodes were homogenized in RPMI-1640 culture medium with 10% fetal bovine serum, 100 U/ml penicillin/streptomycin, 2 mM L-glutamine, 1x MEM non-essential amino acids (Gibco, USA), 1 mM sodium pyruvate (Corning, USA), 10 mM HEPES (Fisher Scientific, USA), and 40 µM 2-mercaptoethanol (Sigma Aldrich) in gentleMACS C tubes (Miltenyi, USA). 0.5x10^6^ cells were incubated for 4 h with 20 ng/ml phorbol 12-myristate 13-acetate (PMA), 1 µg/ml ionomycin (Sigma Aldrich), and 3 µg/ml brefeldin A (Invitrogen, USA).

Subsequently, cells were washed with stain buffer containing bovine serum albumin (BD Pharmingen, USA) and stained with anti-mouse CD4 (clone GK1.5) and anti-mouse CD8a (clone 53-6.7, BioLegend, USA). Cells were then fixed, washed, and stained with anti-mouse IFN-γ antibody (clone XMG1.2, BioLegend), anti-mouse IL-4 antibody (clone BVD4-1D11, Miltenyi), or anti-mouse IL-17A antibody (clone TC11-18H10.1, BioLegend). Cells were analyzed on a Novocyte 3005 flow cytometer (Acea Biosciences, USA). The following gating strategy was used in FlowJo software v.10.6.0 (FlowJo, USA): size (FSC-H vs. SSC-H) → single cells (FSC-H vs. FSC-A) → CD8a+ vs CD4+ → IFN-γ+ (Th1), IL-4+ (Th2), or IL-17A+ (Th17) of the CD4+CD8- population.

### IL-33-Induced Spontaneous Dermatitis

IL-33 transgenic (IL-33tg) mice spontaneously develop itchy dermatitis symptoms after 8 weeks of age, with prominent skin lesions around the eyes and the base of the tail (16, 30). For a prophylactic treatment study, 5-week-old IL-33tg mice were randomized to the following groups: vehicle cream, 1.5% w/w ruxolitinib cream, 0.05% w/w clobetasol cream and non-transgenic littermate control treated with vehicle cream. Mice were dosed twice daily for 7 weeks on the skin around the eyes and tail base.

In a therapeutic treatment paradigm, 12 to 14-week-old IL-33tg mice, with confirmed dermatitis, were randomized to; vehicle cream b.i.d., 1.5% w/w ruxolitinib cream b.i.d., 0.05% w/w betamethasone cream twice a week, and non-transgenic littermate control treated with vehicle cream b.i.d., and treated to 24 weeks of age.

Mice were recorded by video camera for 30 min to quantify excessive scratching and grooming behavior. Hind limb scratching frequency and fore limb grooming were calculated as a cumulative count in 5 min from the video recording.

At study end, skin from the right eyelid was formalin fixed for histology. Skin from the left eyelid was collected for RNA analysis.

### Histological Analysis

Paraffin sections were stained with hematoxylin-eosin and scored by a pathologist blinded to the treatment groups. Histopathology of ear biopsies was graded based on the extent of inflammation, inflammatory cell infiltration, hyperplasticity, and presence of crusting, ulcers, and erosions. Skin samples from IL-33tg mice were graded based on the condition of the epidermis, keratinization, inflammation, and mast cell activation.

### *Ex Vivo* Human Skin

Healthy human skin explant cultures from 4 unrelated donors were dermatomed to a thickness of 750 µm and sectioned into approximately 1 cm^2^ pieces. The explants were mounted onto 0.6 cm^2^ static Franz cells for topical drug dosing at 40 h and 16 h prior to stimulation. The skin samples were positioned with the epidermis facing up, with the donor compartment of the Franz cell clamped in place to prevent lateral migration of ruxolitinib cream around the tissue. Following tissue pre-treatment with ruxolitinib cream, the Th2 stimulation was performed once at time 0, as described (31). The treatment groups included; 1) unstimulated and untreated, 2) Th2 stimulation and untreated, 3) Th2 stimulation treated with vehicle cream and 4) Th2 stimulation treated with 1.5% w/w ruxolitinib cream. The explants were harvested at 6, 24, and 48 h post-stimulation.

### Transcriptomic Pathway Analysis

Total mouse (50 ng) and total human (100 ng) RNA were processed using the nCounter autoimmune profiling codeset (770 genes) or the neuropathology profiling codeset (770 genes) (Nanostring, USA), according to the manufacturer’s protocol. After an 18 h hybridization, the samples were run on an nCounter SPRINT Profiler (Nanostring, USA). Data was analyzed using nSolver 4.0 Advanced Analysis software (Nanostring, USA). P-values were adjusted using the Benjamini-Yekutieli false discovery rate method.

### Statistical Analysis

Statistical analysis was performed using Graphpad Prism 7.04 (Graphpad Software Inc, USA). Differences between groups were assessed by Kruskal-Wallis with Dunn’s post-hoc test for non-parametric data, or ANOVA with Dunnett’s post-hoc test for parametric data sets.

## Results

### Ruxolitinib Cream Inhibits TSLP-Induced Dermatitis by Modulating Multiple Inflammatory Pathways

**Figure 1:**
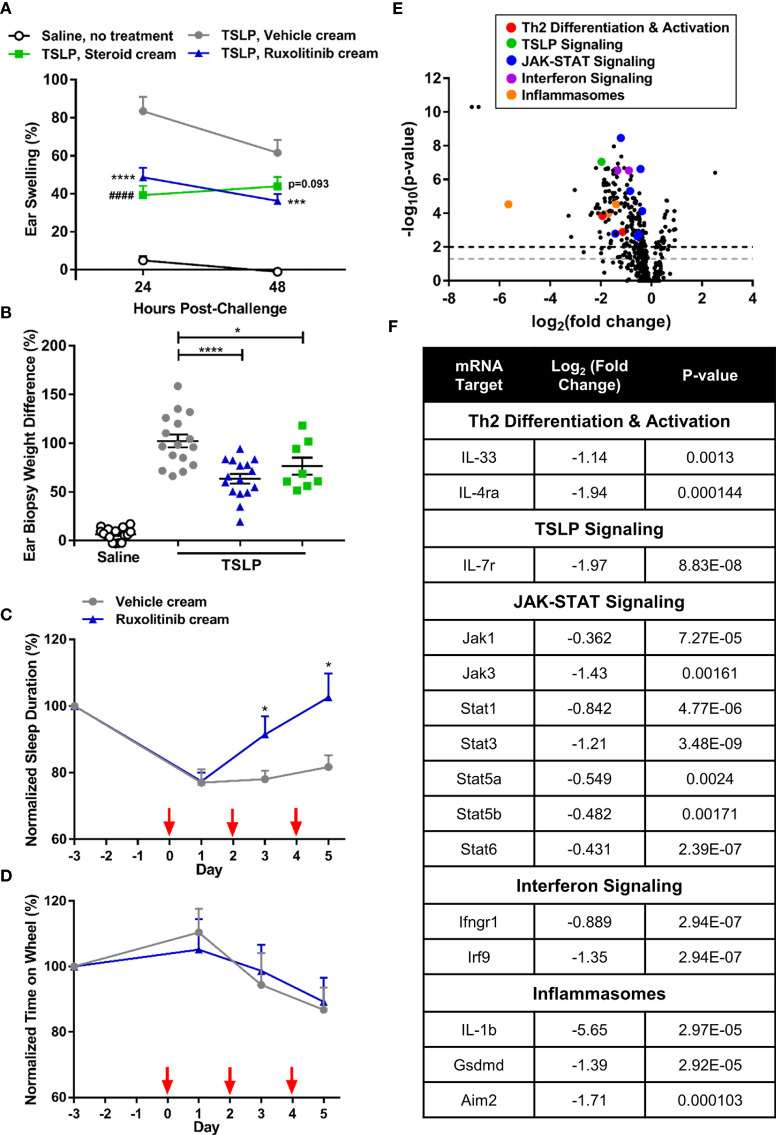
Ruxolitinib cream decreased inflammation and restored sleep duration in the acute thymic stromal lymphopoietin (TSLP)-induced dermatitis mouse model. **(A)** Repeated intradermal TSLP challenge induced acute ear swelling, which was significantly abrogated by ruxolitinib cream treatment. **(B)** Ruxolitinib cream efficacy was confirmed by a significant decrease in ear biopsy weight (% increase between right challenged ear and left control ear) at study termination. N = 16 mice per group. **(C)** Treatment with ruxolitinib cream restored sleep duration (sum of time points in a 24 h period where detectable motion ≤ 3) to baseline levels without causing sedation **(D)**, as shown by unaltered wheel activity (sum of time spent on wheel during a 24 h period) across groups. Red arrows indicate TSLP immunizations. N = 10 mice per group **(C, D)**. **(E)** Nanostring ear skin RNA analysis revealed differential gene expression between ruxolitinib cream and vehicle cream (baseline) treatment. Points above the gray and black dashed lines have adjusted p-values <0.05 and <0.01, respectively. **(F)** Inflammatory genes from multiple pathways were downregulated with ruxolitinib cream treatment. N = 8 mice per group. Data represents mean + SEM. **(A)** ***p < 0.001, ****p < 0.0001, vehicle vs. ruxolitinib. ^####^p<0.0001, vehicle vs. steroid. **(B, C)** *p < 0.05, ****p < 0.0001.

Ruxolitinib cream significantly ameliorated TSLP-induced ear swelling (p<0.001) and tissue biopsy weight (p<0.05) compared to the vehicle cream (**Figures 1A, B**). Experimentally induced dermatitis was associated with disruption of the normal, healthy, sleep-wake cycle in freely moving mice within their home cages. After 3 days of dosing, ruxolitinib cream significantly improved the sleep pattern, leading to fully normalized sleep on day 5 (**Figure 1C**). Importantly, ruxolitinib application did not alter voluntary motor activity, such as running on a wheel (**Figure 1D**), suggesting the reversal of sleep disturbance was not associated with a drug-induced sedation. Transcriptomic analysis revealed 1.5% w/w ruxolitinib cream treatment differentiated from vehicle-dosed animals (**Figure 1E**). Ruxolitinib treatment resulted in a significant downregulation in pro-inflammatory and JAK-STAT signaling pathway genes, including IL-33 (*Il33*), IL-4 receptor alpha chain (*Il4ra*), IL-7 receptor (*Il7r*), interferon gamma receptor 1 (*Ifngr1*), interferon regulatory factor 9 (*Irf9*), *IL-1 beta (Il1b)*, gasdermin (*Gsdmd*), interferon-inducible protein Aim2 (*Aim2*), and *Jak1*, *Jak3*, *Stat1*, *Stat3*, *Stat5a*, *Stat5b*, and *Stat6* (**Figure 1F**).

### Ruxolitinib Cream Ameliorates Chronic FITC-induced Dermatitis *Via* Modulation Inflammatory T-Cell Subsets

**Figure 2:**
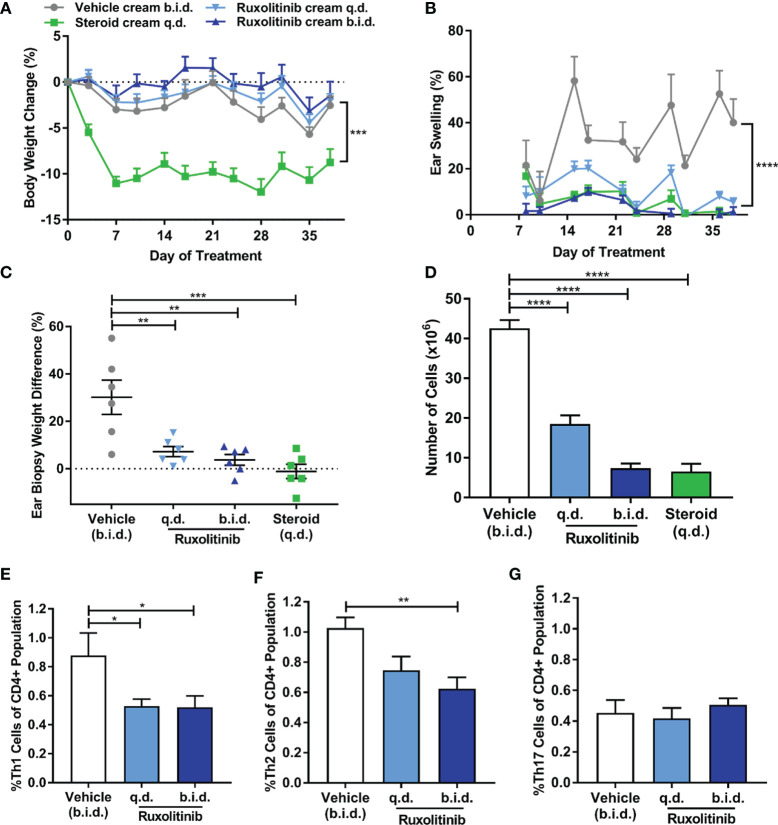
Ruxolitinib cream reduced chronic ear swelling and modulated inflammatory T-cell subsets in the fluorescein isothiocyanate (FITC)-induced dermatitis model. **(A)** Repeated FITC challenge did not induce inflammation-induced cachexia. Steroid (betamethasone) cream was poorly tolerated as indicated by the significant body weight loss. **(B)** Repeated FITC challenge resulted in chronic, non-resolving ear swelling in the vehicle group. **(C)** Ruxolitinib cream dose-dependently ameliorated ear swelling, which was confirmed by a significant decrease in ear biopsy weight (% increase between right challenged ear and left control ear) compared to the vehicle group at study termination. **(D)** Ruxolitinib cream treatment inhibited FITC-induced immune expansion in the auricular lymph node and resulted in lower proportions of Th1 **(E)** and Th2 **(F)** cells compared to vehicle. **(G)** Th17 cell proportions were not affected by ruxolitinib cream treatment in this model. Data represents mean + SEM. N = 6 mice per group. **(A)** ***p < 0.001, vehicle vs. steroid. **(B)** ****p < 0.0001, vehicle vs. ruxolitinib cream q.d. and b.i.d. **(C–G)** *p < 0.05, **p < 0.01, ***p < 0.001, ****p < 0.0001.

Ruxolitinib cream was not associated with drug-induced cachexia (p<0.001) (**Figure 2A**). FITC challenge resulted in chronic, non-resolving ear swelling in the vehicle group, while ruxolitinib cream dose-dependently ameliorated the disease. At study termination, the ruxolitinib cream b.i.d. and q.d. treated groups exhibited 1.4% and 5.5% ear swelling only, which was in contrast to 40% ear swelling in the vehicle group (p<0.0001) (**Figure 2B**). Ruxolitinib cream also significantly (p<0.01) decreased ear biopsy weights (**Figure 2C**).

FITC-induced dermatitis was associated with lymphocyte expansion in the auricular lymph nodes of the vehicle-treated mice, while ruxolitinib cream dose-dependently inhibited this response (p<0.0001) (**Figure 2D**). The proportion of Th1 cells (p<0.05) (**Figure 2E**) and Th2 cells (p<0.01) (**Figure 2F**) was significantly reduced in the ruxolitinib cream treated animals. No significant difference in the proportion of Th17 cells between groups was observed (**Figure 2G**). Cells from the steroid-treated group were unable to be analyzed due to low cell count and viability.

### Ruxolitinib Cream Abrogates Pruritus-Induced Behaviors and Reduces Dermatitis Pathology

**Figure 3:**
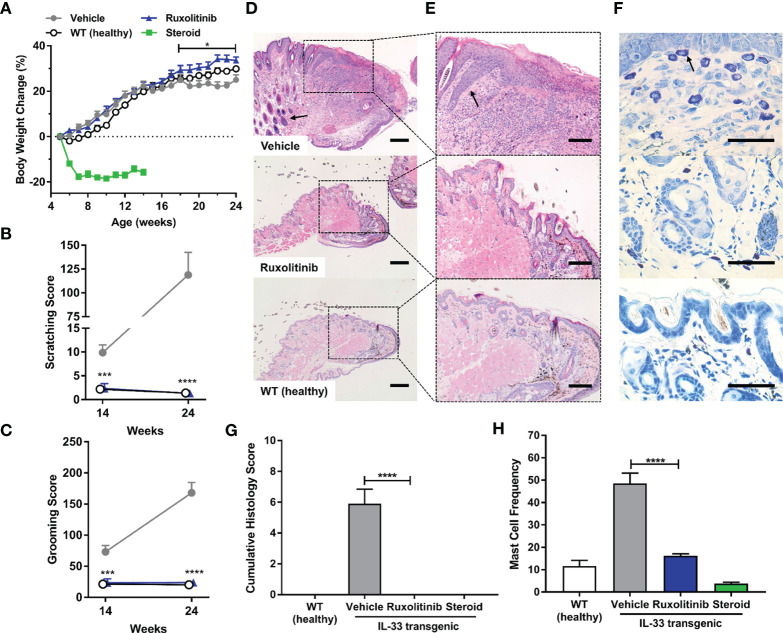
Prophylactic ruxolitinib dosing regimen ameliorated pruritus-induced behaviors and skin histopathology in IL-33tg mice. **(A)** In the IL-33tg dermatitis model, prophylactic treatment with ruxolitinib cream significantly ameliorated body weight loss compared to the vehicle cream group. Steroid (clobetasol) cream was poorly tolerated, as evident in significant body weight loss that required euthanasia at study midpoint. All mice in the steroid group were therefore euthanized at study midpoint, and their tissues were processed for histology. IL-33tg mice treated with vehicle cream exhibited abnormal scratching **(B)** and grooming behavior **(C)**, while prophylactic treatment with topical ruxolitinib significantly ameliorated these behaviors at both study midpoint and endpoint. Eyelid skin samples were stained with hematoxylin and eosin and visualized under **(D)** 20x (scale bar = 200 µm) and **(E)** 50x (scale bar = 100 µm) magnification. The black arrow in vehicle panel **(D)** indicates lymphocyte infiltration. The black arrow in vehicle panel **(E)** indicates epidermal acanthosis. **(F)** Toluidine blue-stained mast cells were assessed under 200x magnification (scale bar = 50 µm). The black arrow in vehicle panel **(F)** indicates mast cell granule staining. Prophylactic application of ruxolitinib cream significantly reduced mast cell frequency **(G)** and cumulative histology score **(H)**. Data represents mean + SEM. N = 10 in vehicle and ruxolitinib groups. N = 5 in WT and steroid groups. *p < 0.05, ***p < 0.001, ****p < 0.0001, vehicle vs. ruxolitinib.

To evaluate the effectiveness of ruxolitinib cream on an acute inflammatory lesion formation, treatment was initiated prior to appearance of overt dermatitis symptoms in the spontaneous, non-remitting, IL-33tg model. Ruxolitinib cream treatment was not associated with any adverse effects and significantly ameliorated body weight loss compared to vehicle cream (p<0.05). In contrast, clobetasol cream was poorly tolerated and caused rapid wasting symptoms including significant body weight loss that required early termination due to humane endpoints (**Figure 3A**). Vehicle-treated IL-33tg mice exhibited abnormal scratching and grooming behavior that worsened over time. Prophylactic treatment with ruxolitinib cream normalized scratching and grooming to baseline levels observed in healthy wild-type littermate controls (p<0.001) (**Figures 3B, C**). Prophylactic application of ruxolitinib cream abrogated all histological signs of skin inflammation, including lymphocytic cell infiltrates (**Figures 3D, E, G**), and reduced mast cell frequencies to normal levels (p<0.0001) (**Figures 3F, H**). The improvement of skin condition was underscored by a significant decrease in cumulative histology score (p<0.0001) (**Figure 3G**).

**Figure 4:**
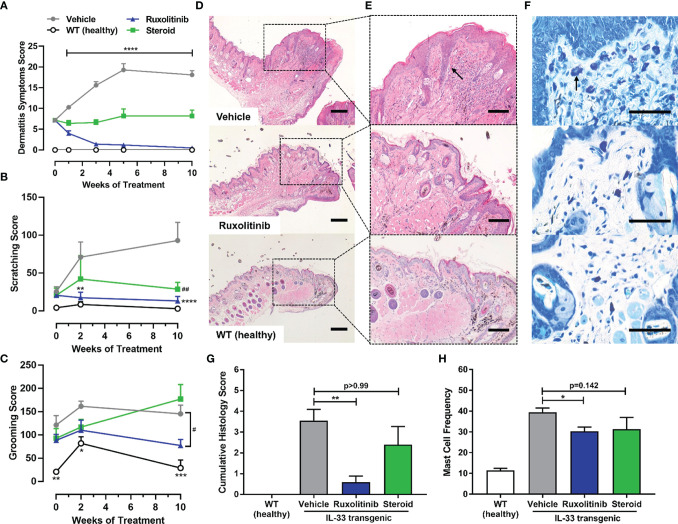
Therapeutic ruxolitinib dosing regimen resolved dermatitis symptoms, pruritus-induced behaviors and skin histopathology in IL-33tg mice. **(A)** Therapeutic ruxolitinib cream treatment significantly resolved dermatitis symptoms in IL-33tg mice, while steroid (betamethasone) cream only prevented further disease progression over time. The vehicle group exhibited abnormal scratching **(B)** and grooming **(C)** behavior. Topical ruxolitinib significantly reduced both scratching and grooming by week 24. Eyelid skin samples were stained with hematoxylin and eosin and visualized under **(D)** 20x (scale bar = 200 µm) and **(E)** 50x (scale bar = 100 µm) magnification. The black arrow in vehicle panel **(E)** indicates epidermal acanthosis. The number of mast cells was scored from toluidine blue stain at 200x magnification (scale bar = 50 µm) **(F)**. The black arrow in vehicle panel **(F)** indicates mast cell granule staining. Therapeutic ruxolitinib cream treatment significantly reduced mast cell frequency **(G)** and cumulative histology score **(H)**. There was no significant difference between the vehicle and steroid group. Data represents mean + SEM. N = 10 in vehicle and ruxolitinib groups. N = 5 in WT and steroid groups. **(A)** ****p < 0.0001, vehicle vs. ruxolitinib. **(B)** **p < 0.01, ****p < 0.0001, vehicle vs. ruxolitinib; ^##^p < 0.01, vehicle vs. steroid. **(C)** *p < 0.05, **p < 0.01, ***p < 0.001, vehicle vs. WT; ^#^p < 0.05, vehicle vs. ruxolitinib. **(D, E)** *p < 0.05, **p < 0.01.

The IL-33tg mouse model was also used to evaluate a therapeutic treatment of established, progressing, dermatitis. Vehicle cream treated IL-33tg mice exhibited time-dependent worsening of dermatitis symptoms (p<0.0001). In contrast, ruxolitinib cream application rapidly reduced dermatitis score after one week of treatment, and after 3 weeks of treatment the scoring was similar to wild-type healthy animals. Steroid cream prevented dermatitis symptoms from progressing, but could not reverse established disease (**Figure 4A**). Similar to the dermatitis score, spontaneous scratching behavior in the vehicle treated animals worsened over time. Again, ruxolitinib cream administration normalized scratch counts (p<0.0001) from the second week of treatment onwards to levels observed in healthy wild-type mice. Steroid cream also ameliorated abnormal scratching behavior (p<0.01), but was numerically inferior to ruxolitinib cream (**Figure 4B**). Spontaneous forelimb grooming behavior was increased in vehicle-treated IL-33tg mice compared to healthy wild-type littermates. Topical ruxolitinib cream administration prevented worsening of this pathological behavior and was statistically significant compared to vehicle treated animals (p<0.05). Notably, steroid treatment did not appear to ameliorate dermatitis-induced grooming behavior (**Figure 4C**). Consistent with the in-life dermatitis and pruritus scoring, histological analysis revealed ruxolitinib cream was highly effective at reducing skin inflammation (p<0.01) and was numerically superior to steroid cream. (**Figures 4D–G**). Ruxolitinib cream also significantly reduced skin tissue mast cell frequency (p<0.05) compared to vehicle treatment (**Figures 4G–H**).

**Figure 5:**
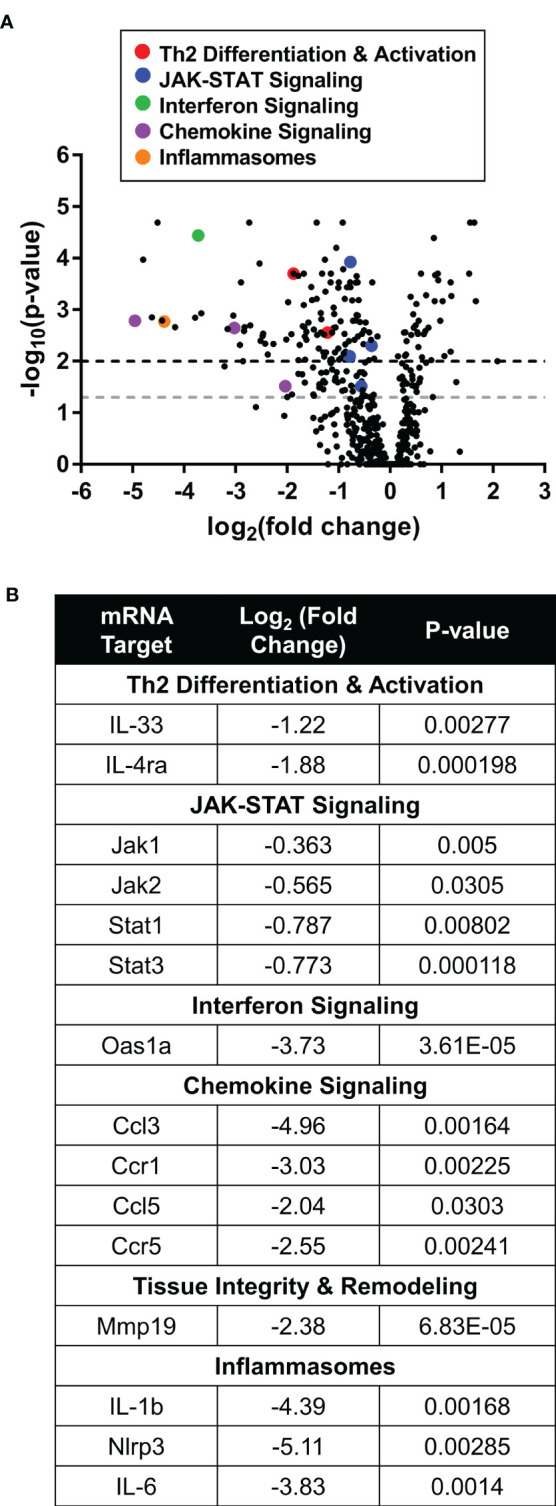
Therapeutic ruxolitinib cream treatment downregulated inflammatory gene expression in IL-33tg mice. **(A)** In the IL-33tg mouse model, Nanostring eyelid skin RNA pathway analysis showed differential gene expression between therapeutic ruxolitinib cream and vehicle cream (baseline) treatment. Points above the gray and black dashed lines have adjusted p-values <0.05 and <0.01, respectively. **(B)** Inflammatory genes from multiple pathways were downregulated with ruxolitinib cream treatment. N = 7 mice per group.

Transcriptomic pathway analysis revealed clear differentiation between the ruxolitinib and vehicle groups (**Figure 5A**). Topically administered ruxolitinib cream downregulated multiple components of the JAK-STAT signaling cascade. In addition, ruxolitinib-mediated efficacy was associated with reduced expression of pro-inflammatory cytokines and chemokines, including IL-33 (*Il33*), IL-4ra (*Il4ra*), interferon-induced 2’-5’-oligoadenylate synthase 1A (*Oas1a*), inflammatory chemokines C-C motif chemokine 3 (*Ccl3*), C-C motif chemokine 5 (*Ccl5*), C-C chemokine receptor type 1 (*Ccr1*), and C-C chemokine receptor 5 (*Ccr5*). Furthermore, the JAK-STAT independent inflammasome pathway was also indirectly modulated, suggesting a broader anti-inflammatory tissue microenvironment is generated after ruxolitinib cream treatment (**Figure 5B**).

### Ruxolitinib Cream Downregulated the Inflammatory Transcriptome in Th2-Stimulated Human Skin

**Figure 6:**
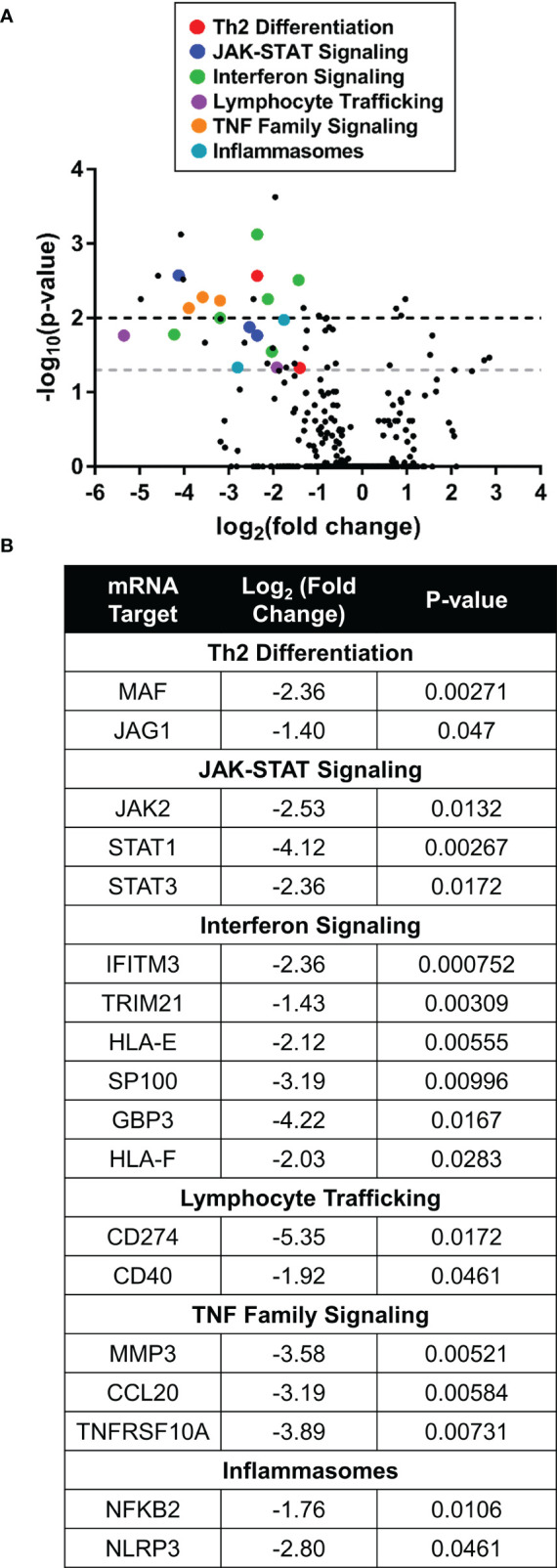
Ruxolitinib cream downregulated expression of inflammatory genes in Th2-stimulated human skin. **(A)** Nanostring RNA pathway analysis showed differential gene expression between ruxolitinib cream and vehicle cream (baseline) treatment in human skin explants stimulated in Th2 conditions. Points above the gray and black dashed lines have adjusted p-values <0.05 and <0.01, respectively. **(B)** Inflammatory genes from multiple pathways were downregulated with ruxolitinib cream treatment. N = 6 donors per treatment.

Differential transcriptomic analysis of Th2-stimulated human skin explants showed statistically significant downregulation of Th2 associated transcripts and decreased JAK-STAT signaling markers in the ruxolitinib cream treatment group compared to vehicle. There was also a downregulation in interferon signaling, lymphocyte trafficking, and TNF family signaling. Inflammasome nuclear factor-kappa-B subunit 2 (*NFKB2*) was also downregulated in response to ruxolitinib (**Figures 6A, B**).

## Discussion

The JAK-STAT pathway has been implicated as a key driver of several inflammatory skin diseases, including AD (20). Targeted pathway modulation *via* the development of JAK inhibitors has uncovered a novel therapeutic opportunity for disease treatment. Recently, topical JAK inhibitors have demonstrated favorable efficacy, safety, and tolerability in clinical trials of patients with AD (27–29).

Ruxolitinib is a potent and selective JAK1/2 inhibitor. Oral ruxolitinib is currently approved for the treatment of patients with myeloproliferative neoplasms (32) and has shown efficacy in treating steroid-refractory graft-versus-host disease (GvHD) (33, 34). A phase 3 study is on-going for the use of oral ruxolitinib in steroid-refractory GvHD (NCT03112603). Ruxolitinib cream is currently being evaluated in clinical trial evaluation for the treatment of mild-to-moderate AD (NCT03745638, NCT03920852 and NCT03745651). Additionally, ruxolitinib cream efficacy has been evaluated in lichen planus, an inflammatory skin condition marked by an itchy rash (NCT03697460).

Ruxolitinib cream was evaluated in multiple experimentally induced models of dermatitis. In one model, dermal inflammation was evoked by TSLP injections. Aberrant TSLP signaling through JAK1/2 has been associated with AD (35, 36). Ruxolitinib cream ameliorated dermatitis symptoms by modulating the transcription of genes directly involved in TSLP signaling, such as IL-7 receptor (*Il7r*) and JAK-STATs (37–39). Levels of interferons are known to increase during the progression of AD from acute to chronic form (40, 41), and elevated transcript levels of IFNγ receptor 1 (*Ifngr1*) and interferon regulatory factor 9 (*Irf9*) were observed in the TSLP-challenged vehicle group. Activation of inflammasomes can lead to an exacerbation of AD (42, 43).

The itch-scratch cycle of AD promotes cutaneous lesion formation and mechanical damage to the epithelial barrier, further enhancing skin inflammation. It is conceivable that by inhibiting TSLP downstream signaling, ruxolitinib cream breaks the itch-scratch cycle allowing for restoration of epithelial barrier, integrity, consequently lowering the expression of JAK-dependent interferon signaling and JAK-independent inflammasome pathway. Notably, ruxolitinib treatment normalized mouse sleep pattern in parallel to resolving skin dermatitis. Sleep disturbance due to pruritus is a debilitating AD symptom (44, 45). Ruxolitinib cream inhibited pruritus without adversely affecting normal voluntary activity, such as wheel running (46, 47).

Repeated FITC challenge resulted in unresolvable ear swelling, consistent with previously published observations (48). Increased infiltration of innate lymphoid cells, a hallmark of AD (49, 50), was also observed. Administration of ruxolitinib cream dose-dependently reduced inflammatory swelling. Efficacy was associated with a significant reduction in total immune cell infiltrates and Th2 and Th1 lymphocytes within the draining auricular lymph node.

Elevated IL-33 expression is observed in AD lesions and blood (10, 51). Following IL-33 binding to its cognate receptor, downstream signaling is dependent on the JAK1/2 heterodimer (12, 13). The activation of IL-33/ST2 signaling and its interaction with primary sensory neurons is a critical component of AD pruritus (15). In mice, overexpression of skin-specific IL-33 leads to AD-like inflammation through activation of Th2 cells, innate lymphoid cells (ILC)2, and mast cells (16, 30, 52). We employed the IL-33tg murine model to test ruxolitinib cream efficacy using both prophylactic and therapeutic dosing regimens. Anti-pruritic efficacy of ruxolitinib was consistent with previous results observed with tofacitinib, a pan-JAK inhibitor that decreased scratching bouts in NC/Nga mice sensitized with dust mite allergen (53). Similarly, ruxolitinib cream provided clinically meaningful reduction in itch in the phase 2 clinical trial in patients with AD (29). In addition to ILC2s, IL-33 promotes mast cell activation, adhesion, migration and maturation (15, 51). IL-33 also indirectly impacts the skin barrier integrity (54). Ruxolitinib cream significantly reduced mast cell frequency as compared to vehicle-only cream and ameliorated skin histopathology, while steroid treatment did not significantly improve cumulative histology score. Ruxolitinib treatment also downregulated *Mmp19* expression, a mechanism which facilitates migration of lymphocytes into inflamed epidermis (55).

Analysis of Th2-stimulated human skin explants corroborated the murine models of dermatitis findings. Inflamed human skin treated with ruxolitinib cream exhibited patterns of differential transcriptomic expression similar to those observed in the mouse dermatitis models, including modulation of the JAK-STAT pathway, interferon signaling, and inflammasome. Consistency of transcriptomic data suggest the translational validity of the animal models.

Atopic dermatitis pathophysiology encompasses skin barrier dysfunction, acute and chronic inflammation, as well as pruritus. In multiple preclinical models of dermatitis, topically applied ruxolitinib cream significantly ameliorated both pathogenic itch, as evaluated in the IL-33tg mice, and dermal inflammation. Taken together, this data strengthens the scientific rationale for topical JAK-STAT pathway inhibition for the treatment of AD.

## Data Availability Statement

The raw data supporting the conclusions of this article will be made available by the authors, without undue reservation.

## Ethics Statement

The animal studies were reviewed and approved by The Institutional Animal Care and Use Committee (IACUC), Incyte.

## Author Contributions

MS and BF contributed equally to performance of the experiments and manuscript drafting. AC performed the Vium data analysis and wrote sections of the manuscript. MP contributed to the development of the TSLP-induced dermatitis model. MS and PS contributed to conception and design of the studies. All authors contributed to the article and approved the submitted version.

## Funding

This study was funded by Incyte Corporation (Wilmington, DE). The funder was not involved in the study design, collection, analysis, interpretation of data, the writing of this article or the decision to submit it for publication.

## Conflict of Interest

All authors are employees and shareholders of Incyte Corporation.

## References

[1] Tsakok T Woolf R Smith CH Weidinger S Flohr C. Atopic dermatitis: the skin barrier and beyond. Br J Dermatol (2019) 180(3):464–74. 10.1111/bjd.16934 29969827

[2] Weidinger S Beck LA Bieber T Kabashima K Irvine AD. Atopic dermatitis. Nat Rev Dis Primers (2018) 4(1):1. 29930242 10.1038/s41572-018-0001-z

[3] Jachiet M Bieuvelet S Argoud AL Vallee M Zinai S Lejeune FX. Sleep disturbance in atopic dermatitis: a case-control study using actigraphy and smartphone-collected questionnaires. Br J Dermatol (2020). 183(3):577–9. 10.1111/bjd.19058 32320479

[4] Cork MJ Danby SG Ogg GS. Atopic dermatitis epidemiology and unmet need in the United Kingdom. J Dermatolog Treat (2020) 31(8):801–09. 10.1080/09546634.2019.1655137 PMC7573657 31631717

[5] Welsch K Holstein J Laurence A Ghoreschi K. Targeting JAK/STAT signalling in inflammatory skin diseases with small molecule inhibitors. Eur J Immunol (2017) 47(7):1096–107. 10.1002/eji.201646680 28555727

[6] Seavey MM Dobrzanski P. The many faces of Janus kinase. Biochem Pharmacol (2012) 83(9):1136–45. 10.1016/j.bcp.2011.12.024 22209716

[7] Liongue C Sertori R Ward AC. Evolution of Cytokine Receptor Signaling. J Immunol (2016) 197(1):11–8. 10.4049/jimmunol.1600372 27317733

[8] Seif F Khoshmirsafa M Aazami H Mohsenzadegan M Sedighi G Bahar M. The role of JAK-STAT signaling pathway and its regulators in the fate of T helper cells. Cell Commun Signal (2017) 15(1):23. 28637459 10.1186/s12964-017-0177-y PMC5480189

[9] Howell MD Fitzsimons C Smith PA. JAK/STAT inhibitors and other small molecule cytokine antagonists for the treatment of allergic disease. Ann Allergy Asthma Immunol (2018) 120(4):367–75. 10.1016/j.anai.2018.02.012 29454096

[10] Howell MD Kuo FI Smith PA. Targeting the Janus Kinase Family in Autoimmune Skin Diseases. Front Immunol (2019) 10:2342. 31649667 10.3389/fimmu.2019.02342 PMC6794457

[11] Akdis CA Arkwright PD Bruggen MC Busse W Gadina M Guttman-Yassky E. Type 2 immunity in the skin and lungs. Allergy (2020) 75(7):1582–605. 10.1111/all.14318 32319104

[12] Pinto SM Subbannayya Y Rex DAB Raju R Chatterjee O Advani J. A network map of IL-33 signaling pathway. J Cell Commun Signal (2018) 12(3):615–24. 10.1007/s12079-018-0464-4 PMC6039344 29705949

[13] Alvarez F Fritz JH Piccirillo CA. Pleiotropic Effects of IL-33 on CD4(+) T Cell Differentiation and Effector Functions. Front Immunol (2019) 10:522. 30949175 10.3389/fimmu.2019.00522 PMC6435597

[14] Griesenauer B Paczesny S. The ST2/IL-33 Axis in Immune Cells during Inflammatory Diseases. Front Immunol (2017) 8:475. 28484466 10.3389/fimmu.2017.00475 PMC5402045

[15] Chan BCL Lam CWK Tam LS Wong CK. IL33: Roles in Allergic Inflammation and Therapeutic Perspectives. Front Immunol (2019) 10:364. 30886621 10.3389/fimmu.2019.00364 PMC6409346

[16] Imai Y Yasuda K Sakaguchi Y Haneda T Mizutani H Yoshimoto T. Skin-specific expression of IL-33 activates group 2 innate lymphoid cells and elicits atopic dermatitis-like inflammation in mice. Proc Natl Acad Sci USA (2013) 110(34):13921–6. 10.1073/pnas.1307321110 PMC3752227 23918359

[17] Brandt EB Sivaprasad U. Th2 Cytokines and Atopic Dermatitis. J Clin Cell Immunol (2011) 2(3):110. 10.4172/2155-9899.1000110 PMC3189506 21994899

[18] Wilson SR The L Batia LM Beattie K Katibah GE McClain SP. The epithelial cell-derived atopic dermatitis cytokine TSLP activates neurons to induce itch. Cell (2013) 155(2):285–95. 10.1016/j.cell.2013.08.057 PMC4041105 24094650

[19] Shi Z Jiang W Wang M Wang X Li X Chen X. Inhibition of JAK/STAT pathway restrains TSLP-activated dendritic cells mediated inflammatory T helper type 2 cell response in allergic rhinitis. Mol Cell Biochem (2017) 430(1–2):161–9. 10.1007/s11010-017-2963-7 28214951

[20] Cotter DG Schairer D Eichenfield L. Emerging therapies for atopic dermatitis: JAK inhibitors. J Am Acad Dermatol (2018) 78(3 Suppl 1):S53–62. 10.1016/j.jaad.2017.12.019 29248518

[21] Damsky W King BA. JAK inhibitors in dermatology: The promise of a new drug class. J Am Acad Dermatol (2017) 76(4):736–44. 10.1016/j.jaad.2016.12.005 PMC6035868 28139263

[22] Yosipovitch G Rosen JD Hashimoto T. Itch: From mechanism to (novel) therapeutic approaches. J Allergy Clin Immunol (2018) 142(5):1375–90. 10.1016/j.jaci.2018.09.005 30409247

[23] Suga H Sato S. Novel topical and systemic therapies in atopic dermatitis. Immunol Med (2019) 42(2):84–93. 31318324 10.1080/25785826.2019.1642727

[24] Lynde CW Bergman J Fiorillo L Guenther L Keddy-Grant J Landells I. Clinical Insights About Topical Treatment of Mild-to-Moderate Pediatric and Adult Atopic Dermatitis. J Cutan Med Surg (2019) 23(3_suppl):3S–13S. 10.1177/1203475419843108 30965012

[25] Rodrigues MA Torres T. JAK/STAT inhibitors for the treatment of atopic dermatitis. J Dermatolog Treat (2020) 31(1):33–40. 30703333 10.1080/09546634.2019.1577549

[26] Bissonnette R Papp KA Poulin Y Gooderham M Raman M Mallbris L. Topical tofacitinib for atopic dermatitis: a phase IIa randomized trial. Br J Dermatol (2016) 175(5):902–11. 10.1111/bjd.14871 27423107

[27] Dhillon S. Delgocitinib: First Approval. Drugs (2020) 80(6):609–15. 10.1007/s40265-020-01291-2 32166597

[28] Kim BS Howell MD Sun K Papp K Nasir A Kuligowski ME. Treatment of atopic dermatitis with ruxolitinib cream (JAK1/JAK2 inhibitor) or triamcinolone cream. J Allergy Clin Immunol (2020) 145(2):572–82. 10.1016/j.jaci.2019.08.042 31629805

[29] Kim BS Sun K Papp K Venturanza M Nasir A Kuligowski ME. Effects of ruxolitinib cream on pruritus and quality of life in atopic dermatitis: Results from a phase 2, randomized, dose-ranging, vehicle- and active-controlled study. J Am Acad Dermatol (2020) 82(6):1305–13. 10.1016/j.jaad.2020.02.009 32057960

[30] Imai Y Yasuda K Nagai M Kusakabe M Kubo M Nakanishi K. IL-33-Induced Atopic Dermatitis-Like Inflammation in Mice Is Mediated by Group 2 Innate Lymphoid Cells in Concert with Basophils. J Invest Dermatol (2019) 139(10):2185–94 e3. 31121178 10.1016/j.jid.2019.04.016

[31] Neil JE Brown MB Williams AC. Human skin explant model for the investigation of topical therapeutics. Sci Rep (2020) 10(1):21192. 33273665 10.1038/s41598-020-78292-4 PMC7712775

[32] Bryan JC Verstovsek S. Overcoming treatment challenges in myelofibrosis and polycythemia vera: the role of ruxolitinib. Cancer Chemother Pharmacol (2016) 77(6):1125–42. 10.1007/s00280-016-3012-z PMC4882345 27017614

[33] Jagasia M Perales MA Schroeder MA Ali H Shah NN Chen YB. Ruxolitinib for the treatment of steroid-refractory acute GVHD (REACH1): a multicenter, open-label phase 2 trial. Blood (2020) 135(20):1739–49. 10.1182/blood.2020004823 PMC7229262 32160294

[34] Zeiser R von Bubnoff N Butler J Mohty M Niederwieser D Or R. Ruxolitinib for Glucocorticoid-Refractory Acute Graft-versus-Host Disease. N Engl J Med (2020) 382(19):1800–10. 10.1056/NEJMoa1917635 32320566

[35] Kim BS Siracusa MC Saenz SA Noti M Monticelli LA Sonnenberg GF. TSLP elicits IL-33-independent innate lymphoid cell responses to promote skin inflammation. Sci Transl Med (2013) 5(170):170ra16. 10.1126/scitranslmed.3005374 PMC3637661 23363980

[36] Zhong J Sharma J Raju R Palapetta SM Prasad TS Huang TC. TSLP signaling pathway map: a platform for analysis of TSLP-mediated signaling. Database (Oxford) (2014) 2014:bau007. 24573880 10.1093/database/bau007 PMC3935308

[37] Han NR Oh HA Nam SY Moon PD Kim DW Kim HM. TSLP induces mast cell development and aggravates allergic reactions through the activation of MDM2 and STAT6. J Invest Dermatol (2014) 134(10):2521–30. 10.1038/jid.2014.198 24751726

[38] Durum SK. IL-7 and TSLP receptors: twisted sisters. Blood (2014) 124(1):4–5. 24993875 10.1182/blood-2014-05-574327

[39] Rochman Y Kashyap M Robinson GW Sakamoto K Gomez-Rodriguez J Wagner KU. Thymic stromal lymphopoietin-mediated STAT5 phosphorylation via kinases JAK1 and JAK2 reveals a key difference from IL-7-induced signaling. Proc Natl Acad Sci USA (2010) 107(45):19455–60. 10.1073/pnas.1008271107 PMC2984176 20974963

[40] David Boothe W Tarbox JA Tarbox MB. Atopic Dermatitis: Pathophysiology. Adv Exp Med Biol (2017) 1027:21–37. 29063428 10.1007/978-3-319-64804-0_3

[41] Spergel JM Mizoguchi E Oettgen H Bhan AK Geha RS. Roles of TH1 and TH2 cytokines in a murine model of allergic dermatitis. J Clin Invest (1999) 103(8):1103–11. 10.1172/JCI5669 PMC408277 10207161

[42] Sa DC Festa CN. Inflammasomes and dermatology. Bras Dermatol (2016) 91(5):566–78. 10.1590/abd1806-4841.20165577 PMC5087212 27828627

[43] Abramovits W Rivas Bejarano JJ Valdecantos WC. Role of interleukin 1 in atopic dermatitis. Dermatol Clin (2013) 31(3):437–44. 10.1016/j.det.2013.04.008 23827246

[44] Chang YS Chou YT Lee JH Lee PL Dai YS Sun C. Atopic dermatitis, melatonin, and sleep disturbance. Pediatrics (2014) 134(2):e397–405. 10.1542/peds.2014-0376 25022734

[45] Angelhoff C Askenteg H Wikner U Edell-Gustafsson U. “To Cope with Everyday Life, I Need to Sleep” - A Phenomenographic Study Exploring Sleep Loss in Parents of Children with Atopic Dermatitis. J Pediatr Nurs (2018) 43:e59–65. 10.1016/j.pedn.2018.07.005 30037591

[46] Goh J Ladiges W. Voluntary Wheel Running in Mice. Curr Protoc Mouse Biol (2015) 5(4):283–90. 10.1002/9780470942390.mo140295 PMC4686373 26629772

[47] Manzanares G Brito-da-Silva G Gandra PG. Voluntary wheel running: patterns and physiological effects in mice. Braz J Med Biol Res (2018) 52(1):e7830. 30539969 10.1590/1414-431X20187830 PMC6301263

[48] Li J Li L Zuo H Ke C Yan B Wen H. T-helper type-2 contact hypersensitivity of Balb/c mice aggravated by dibutyl phthalate via long-term dermal exposure. PLoS One (2014) 9(2):e87887. 24498391 10.1371/journal.pone.0087887 PMC3912153

[49] Kim BS. Innate lymphoid cells in the skin. J Invest Dermatol (2015) 135(3):673–8. 10.1038/jid.2014.401 PMC4556524 25339380

[50] Sabat R Wolk K Loyal L Docke WD Ghoreschi K. T cell pathology in skin inflammation. Semin Immunopathol (2019) 41(3):359–77. 10.1007/s00281-019-00742-7 PMC6505509 31028434

[51] Klonowska J Glen J Nowicki RJ Trzeciak M. New Cytokines in the Pathogenesis of Atopic Dermatitis-New Therapeutic Targets. Int J Mol Sci (2018) 19(10):3086. 10.3390/ijms19103086 PMC6213458 30304837

[52] Imai Y. Interleukin-33 in atopic dermatitis. J Dermatol Sci (2019) 96(1):2–7. 31455506 10.1016/j.jdermsci.2019.08.006

[53] Fukuyama T Ehling S Wilzopolski J Baumer W. Comparison of topical tofacitinib and 0.1% hypochlorous acid in a murine atopic dermatitis model. BMC Pharmacol Toxicol (2018) 19(1):37. 29970189 10.1186/s40360-018-0232-3 PMC6029395

[54] Seltmann J Roesner LM von Hesler FW Wittmann M Werfel T. IL-33 impacts on the skin barrier by downregulating the expression of filaggrin. J Allergy Clin Immunol (2015) 135(6):1659–61. 10.1016/j.jaci.2015.01.048 25863977

[55] Beck IM Ruckert R Brandt K Mueller MS Sadowski T Brauer R. MMP19 is essential for T cell development and T cell-mediated cutaneous immune responses. PLoS One (2008) 3(6):e2343. 18523579 10.1371/journal.pone.0002343 PMC2386969

